# An Electret-Augmented Low-Voltage MEMS Electrostatic Out-of-Plane Actuator for Acoustic Transducer Applications

**DOI:** 10.3390/mi11030267

**Published:** 2020-03-04

**Authors:** Chikako Sano, Manabu Ataka, Gen Hashiguchi, Hiroshi Toshiyoshi

**Affiliations:** 1 Institute of Industrial Science, The University of Tokyo, 4-6-1 Komaba, Meguro-ku, Tokyo 153-8505, Japan; 2 Research Institute of Electronics, Shizuoka University, 3-5-1 Johoku, Naka-ku, Hamamatsu, Shizuoka 432-8011, Japan

**Keywords:** electrets, low-voltage-driven, acoustic transducers, SOI-MEMS

## Abstract

Despite the development of energy-efficient devices in various applications, microelectromechanical system (MEMS) electrostatic actuators yet require high voltages to generate large displacements. In this respect, electrets exhibiting quasi-permanent electrical charges allow large fixed voltages to be integrated directly within electrode structures to reduce or eliminate the need of DC bias electronics. For verification, a −40 V biased electret layer was fabricated at the inner surface of a silicon on insulator (SOI) structure facing a 2 μm gap owing to the high compatibility of silicon micromachining and the potassium-ion-electret fabrication method. A −10 V electret-augmented actuator with an out-of-plane motion membrane reached a sound pressure level (SPL) of 50 dB maximum with AC input voltage of $V_{in}=5\;V_{\mathrm{pp}}$ alone, indicating a potential for acoustic transducer usage such as microspeakers. Such devices with electret biasing require only the input signal voltage, thus contributing to reducing the overall power consumption of the device system.

## 1. Introduction

**Figure 1:**
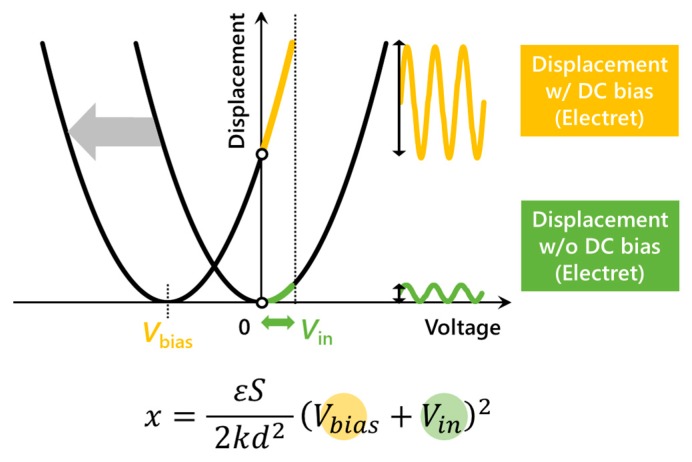
Displacement of MEMS electrostatic actuator augmented by DC biasing from electrets replacing external voltage sources.

High-voltage requirements have long been an issue for electrostatically driven microelectromechanical system (MEMS) devices, especially for actuators which convert electrical signals to significant mechanical motion. For example, MEMS actuators replacing conventional electromagnetic driving components of analog integrated circuits demanded a high-voltage source which originally was unnecessary. Furthermore, MEMS actuators, along with sensors, are key features in internet-of-things (IoT) networks where sensors gather the information and the processed data is feedbacked to the environment through actuator motions. However, IoT modules consisting of actuators with high-voltage or large-current requirements lack overall portability, given that battery sizes generally correspond to their capacities. In the first place, electrostatically driven MEMS actuators, compared to other driving principles such as electromagnetic types, are compatible with these wireless IoT modules in the sense that capacitors do not consume large current which shortens battery lives. This research deals with the remaining high-voltage issue by integrating electret materials into electrostatically driven MEMS actuators. Electret materials exhibiting a quasi-permanent charge can eliminate or reduce the necessity for an externally applied DC voltage. As is shown in Figure 1, with the same AC voltage range, MEMS actuators featuring electrets can generate large displacements that would otherwise require an additional DC bias. The DC biasing is also necessary to match the frequency of the AC driving voltage to the actuation frequency.

However, electret-applicable MEMS actuator structures were limited. Conventional electret fabrication techniques, corona charging method [1] being a common one, were based on charge transfer depending on a discharge in the air gap. Due to their charging setups, fine electrets were formed on a flat surface of a base material but not on faces of silicon micromachined structures such as sidewalls of high-aspect-ratio trench structures which are inevitable components in high performance MEMS electrostatic devices. Devices can be fabricated by attaching electret chips to MEMS devices, but this would interfere the largest feature of MEMS: mass production by batch processing without assembly. Amongst several researches seeking for electret fabrication on micromachined trench structures [2,3], the potassium-ion-electret method [4,5,6] has a high compatibility with silicon MEMS and is used in this research. The electret can be fabricated as a post-processing of silicon micromachining, where the electret potential is formed by applying a DC voltage under high temperature to a thermally grown potassium ion incorporated silicon dioxide.

**Figure 2:**
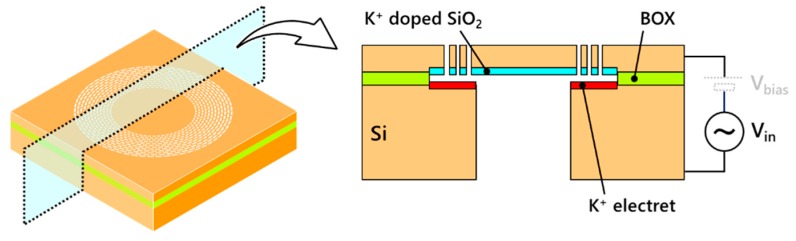
Concept of narrow-gap electret fabrication at inner surface of SOI. Electret augments out-of-plane motion of membrane fabricated on device layer.

In this research, an electret layer is fabricated at the inner surface of a SOI (silicon on insulator)-MEMS device to augment the out-of-plane motion of the membrane structure in the device layer, as shown in Figure 2. The parallel electrodes, one at the device layer and the other at the handle layer of the SOI, face a narrow gap of 2 μm fabricated by removing the sacrificial BOX (buried oxide) layer. Compared to applying electrets on sidewalls of trenches fabricated in the device layer of the SOI [7,8,9], the electret faces a narrower but uniform gap since sacrificial layer etching does not yield any scallops or high-aspect-ratio limitation which existed at gap formation using DRIE (deep reactive ion etching). Subsequently, acoustic characteristics are measured and evaluated for possible application of this out-of-plane membrane structure as acoustic transducers such as microspeakers. Electrostatic microspeakers yet require several tens of volts of DC biasing to reach sufficient acoustic outputs [10,11,12,13,14,15,16,17,18], which leads to large power consumption in the electrical circuits. Although electrets have been applied to speakers in a large scale of centimeters [19,20,21,22,23], microspeakers with electrets were difficult to realize due to the incompatibility of microstructures and the electret fabrication techniques. By decreasing the high-voltage demand by our electret fabrication technique, electrostatic microspeakers would be of practical usage comparatively to electromagnetic ones, of which most of the existing commercial microspeakers are.

First shown in this paper are analytical modeling results of electret application to electrostatic actuators using a parallel plate electrode model for first order approximation. Then, to demonstrate this displacement augmentation by electrets, an out-of-plane membrane actuator is fabricated. Electromechanical and acoustic characteristics of the device are measured and evaluated to verify the DC biasing generated from the electret layer.

## 2. Analytical Modeling of Electret-Augmented Electrostatic Actuators

### 2.1. Electret Potential

**Figure 3:**
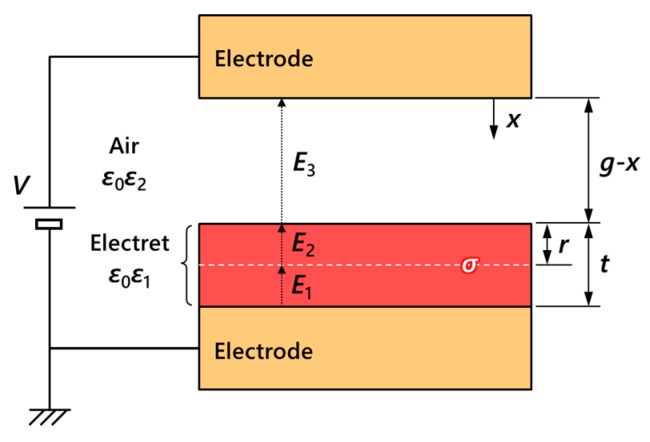
Electrical modeling of gap-closing parallel plate electrodes with electret.

In order to model the potential of an electret formed against a counterpart electrode, a gap-closing parallel plate electrode model shown in Figure 3 is illustrated. A single charge layer with an area charge density of σ exists at distance r from the surface of the electret material with thickness t and a relative dielectric constant of $\varepsilon_{1}$. This electret material faces the counterpart electrode with an initial gap g filled of air with a relative dielectric constant $\varepsilon_{2}$. Electric fields are expressed respectively: $E_{1}$ between the back electrode and the charge layer, $E_{2}$ between the charge layer and the electret surface, and $E_{3}$ for the air gap. Voltage V is applied between the electrodes from an external voltage source. The electric flux density at each interface can be driven from Gauss’ law as

(1) $$\varepsilon_{1}E_{2}-\varepsilon_{1}E_{1}=\frac{\sigma }{\varepsilon_{0}},\;\varepsilon_{2}E_{3}-\varepsilon_{1}E_{2}=0,$$

where $\varepsilon_{0}$ is the dielectric constant of vacuum. Also, from Kirchhoff’s second law is

(2) $$\left(g-x\right)E_{3}+rE_{2}+\left(t-r\right)E_{1}=-V.$$

From Equations (1) and (2), $E_{1}$, $E_{2}$, and $E_{3}$ are expressed as

(3) $$E_{1}=\frac{\varepsilon_{1}\varepsilon_{2}}{\varepsilon_{1}\left(g-x\right)+\varepsilon_{2}t}\left[-\frac{V}{\varepsilon_{1}}-\frac{\sigma \left(\frac{r}{\varepsilon_{1}}+\frac{g-x}{\varepsilon_{2}}\right)}{\varepsilon_{0}\varepsilon_{1}}\right]\;E_{2}=\frac{\varepsilon_{1}\varepsilon_{2}}{\varepsilon_{1}\left(g-x\right)+\varepsilon_{2}t}\left[-\frac{V}{\varepsilon_{1}}+\frac{\sigma \left(t-r\right)}{\varepsilon_{0}\varepsilon_{1}^{2}}\right]\;E_{3}=\frac{\varepsilon_{1}\varepsilon_{2}}{\varepsilon_{1}\left(g-x\right)+\varepsilon_{2}t}\left[-\frac{V}{\varepsilon_{2}}+\frac{\sigma \left(t-r\right)}{\varepsilon_{0}\varepsilon_{1}\varepsilon_{2}}\right].$$

Therefore, the electret potential $V_{e}=\left(g-x\right)\;E_{3}$ formed between the electret and the counterpart electrode at V=0 is

(4) $$V_{e}=\frac{\sigma \left(t-r\right)}{\varepsilon_{0}\left(\varepsilon_{1}+\varepsilon_{2}\frac{t}{g-x}\right)}.$$

When the electret and the counterpart electrode are at a large distance of (g−x)≫t, the potential measured at the electret surface is expressed as

(5) $$V_{e}=\frac{\sigma \left(t-r\right)}{\varepsilon_{0}\varepsilon_{1}}.$$

Meanwhile, the electrostatically driven charges at the upper and the lower electrode, $Q_{U}$ and $Q_{L}$, are respectively expressed as

(6) $$\begin{array}{cc} Q_{U} & =-\varepsilon_{0}\varepsilon_{2}E_{3}S\\ & =S\frac{\varepsilon_{0}\varepsilon_{1}\varepsilon_{2}V+\varepsilon_{2}\sigma \left(t-r\right)}{\varepsilon_{1}\left(g-x\right)+\varepsilon_{2}t}\\ & =S\frac{\varepsilon_{0}\varepsilon_{1}\varepsilon_{2}\left(V-V_{e}\right)}{\varepsilon_{1}\left(g-x\right)+\varepsilon_{2}t}, \end{array}$$

and

(7) $$\begin{array}{cc} Q_{L} & =\varepsilon_{0}\varepsilon_{1}E_{1}S\\ & =-S\frac{\varepsilon_{0}\varepsilon_{1}\varepsilon_{2}V+\sigma \left[\varepsilon_{1}\left(g-x\right)+\varepsilon_{2}r\right]}{\varepsilon_{1}\left(g-x\right)+\varepsilon_{2}t}\\ & =-S\frac{\varepsilon_{0}\varepsilon_{1}\varepsilon_{2}\left(t-r\right)V+\varepsilon_{0}\varepsilon_{1}\left[\varepsilon_{1}\left(g-x\right)+\varepsilon_{2}r\right]V_{e}}{\left(t-r\right)\left[\varepsilon_{1}\left(g-x\right)+\varepsilon_{2}t\right]} \end{array}.$$

### 2.2. Electrostatic Force with Electrets

**Figure 4:**
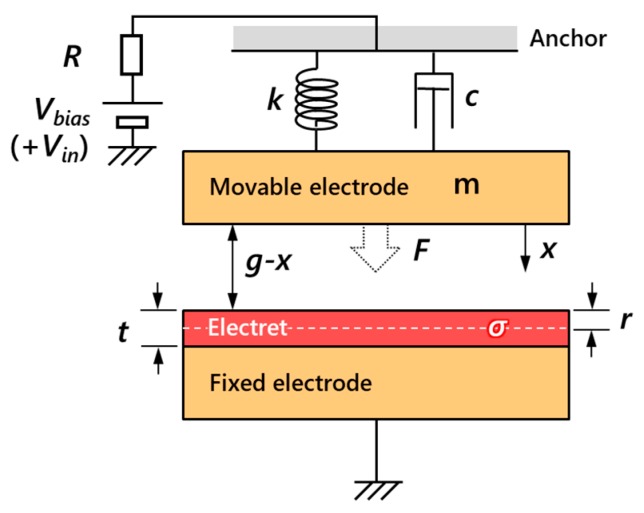
Electromechanical modeling of gap-closing parallel plate electrodes with electret.

The electrostatic force F as a result of the electret potential $V_{e}$ and an external voltage of V is considered with Figure 4 of which the electrical properties correspond to Figure 3. Given the upper movable electrode’s virtual displacement of Δx, the mechanical work ΔW=FΔx done by the movable electrode together with the difference ΔU of the electrostatic energy stored in parallel plate electrodes equals to the electrical work $\Delta W=V\Delta Q_{U}\;\left(=-V\Delta Q_{L}\right)$ done by the voltage source to charge the capacitor against the voltage difference V. This can be expressed as

(8) $$F\Delta x+\Delta U=V\Delta Q_{U}.$$

Since the electrostatic energy stored in each layer in Figure 3 is a product of the energy density and the volume, the total electrostatic energy U(x) is

(9) $$\begin{array}{cc} U\left(x\right) & =\frac{1}{2}\varepsilon_{0}\varepsilon_{1}E_{1}^{2}\left(t-r\right)S+\frac{1}{2}\varepsilon_{0}\varepsilon_{1}E_{2}^{2}rS+\frac{1}{2}\varepsilon_{0}\varepsilon_{2}E_{3}^{2}\left(g-x\right)S\\ & =\frac{1}{2}SV^{2}\frac{\varepsilon_{0}\varepsilon_{1}\varepsilon_{2}}{\varepsilon_{1}\left(g-x\right)+\varepsilon_{2}t}+\frac{1}{2}S\sigma^{2}\frac{\left(t-r\right)\left[\varepsilon_{1}\left(g-x\right)+\varepsilon_{2}r\right]}{\varepsilon_{0}\varepsilon_{1}\left[\varepsilon_{1}\left(g-x\right)+\varepsilon_{2}t\right]}. \end{array}$$

Therefore, the difference of the electrostatic energy ΔU=U(x+Δx)−U(x) owing to the virtual displacement Δx is expressed as

(10) $$\Delta U=\frac{1}{2}S\frac{\varepsilon_{2}\Delta x\left[\varepsilon_{0}^{2}\varepsilon_{1}^{2}V^{2}-\sigma^{2}{\left(t-r\right)}^{2}\right]}{\varepsilon_{0}\left[\varepsilon_{1}\left(g-x\right)+\varepsilon_{2}t\right]\left[\varepsilon_{1}\left(g-x-\Delta x\right)+\varepsilon_{2}t\right]}.$$

On the other hand, $\Delta Q_{U}$ is the electrostatically driven charge generated from the transition of the electric field due to x→x+Δx. Therefore

(11) $$\begin{array}{cc} \Delta Q_{U} & =-\varepsilon_{0}\varepsilon_{2}S\left[E_{3}\left(x+\Delta x\right)-E\left(x\right)\right]\\ & =S\frac{\varepsilon_{1}\varepsilon_{2}\Delta x\left[\varepsilon_{0}\varepsilon_{1}V-\sigma \left(t-r\right)\right]}{\left[\varepsilon_{1}\left(g-x\right)+\varepsilon_{2}t\right]\left[\varepsilon_{1}\left(g-x-\Delta x\right)+\varepsilon_{2}t\right]}. \end{array}$$

Substituting ΔU and $\Delta Q_{U}$ from Equations (10) and (11), Equation (8) yields

(12) $$F\Delta x=\frac{1}{2}S\frac{\varepsilon_{2}\Delta x{\left[\varepsilon_{0}\varepsilon_{1}V-\sigma \left(t-r\right)\right]}^{2}}{\varepsilon_{0}\left[\varepsilon_{1}\left(g-x\right)+\varepsilon_{2}t\right]\left[\varepsilon_{1}\left(g-x-\Delta x\right)+\varepsilon_{2}t\right]},$$

which is transformed into

(13) $$F=\frac{1}{2}S\frac{\varepsilon_{2}{\left[\varepsilon_{0}\varepsilon_{1}V-\sigma \left(t-r\right)\right]}^{2}}{\varepsilon_{0}\left[\varepsilon_{1}\left(g-x\right)+\varepsilon_{2}t\right]\left[\varepsilon_{1}\left(g-x-\Delta x\right)+\varepsilon_{2}t\right]}.$$

With Δx→0, the electrostatic force F with an external voltage of V applied between an electret and its counterpart electrode is expressed as

(14) $$F=\frac{1}{2}S\frac{\varepsilon_{2}{\left[\varepsilon_{0}\varepsilon_{1}V-\sigma \left(t-r\right)\right]}^{2}}{\varepsilon_{0}{\left[\varepsilon_{1}\left(g-x\right)+\varepsilon_{2}t\right]}^{2}}.$$

Using Equation (5), the electrostatic force F can also be expressed using the electret potential $V_{e}$ as

(15) $$F=\frac{1}{2}S\frac{\varepsilon_{0}\varepsilon_{1}^{2}\varepsilon_{2}{\left(V-V_{e}\right)}^{2}}{{\left[\varepsilon_{1}\left(g-x\right)+\varepsilon_{2}t\right]}^{2}}.$$

### 2.3. Parallel Plate Electrode Model with Electrets

Using the electrostatic force F derived in Section 2.2, motions of the gap-closing parallel plate electret model shown in Figure 4 are simulated using an equivalent circuit model. From Newton’s equation of motion, acceleration $\ddot{x}$ of the movable electrode can be expressed as

(16) $$\ddot{x}=\frac{1}{m}\left(F-c\dot{x}-kx\right),$$

where m is the mass, c is the viscous damping coefficient, and k is the spring constant. Equation (16) is solved as a feedback circuit using LTspice as an analog computer, by representing displacement and velocity with voltage, and force with current. 

**Figure 5:**
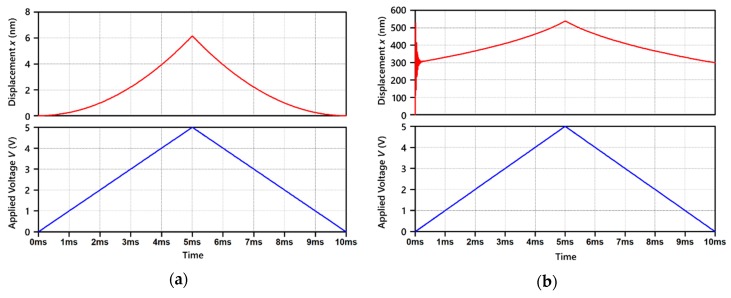
Simulation of static characteristics of gap-closing parallel plate electrode model with electret: (**a**) electret potential $V_{e}=0\;V$; (**b**) electret potential $V_{e}=-30\;V$.

Arbitrary parameters listed in Table 1 are used to simulate and comprehend the motion of parallel plate electrodes with electrets. Since the displacement is sufficiently small compared to the scale of electrodes, stress hardening or softening is negligible and spring constant k is expressed as a fixed value by considering the suspension as a double-clamped beam with its thickness and the length being respectively equal to the diaphragm thickness and the diameter. Spring constant k and damping coefficient c are chosen a priori from the resonance frequency and the Q value of the experimental results, respectively. Figure 5 shows the static characteristics of the movable electrode, where the electret potentials are $V_{e}=0\;V$ in Figure 5a and $V_{e}=-30\;V$ in Figure 5b. While sweeping the voltage V applied to the counterpart electrode of the electret electrode, which is the upper electrode in Figure 3 and Figure 4, from V=0 V to 5 V, a significant difference appeared in the amount of the displacement. The sweep time was set to be sufficiently longer than the period where the mechanical resonance occurs. The displacement difference for $V_{e}=-30\;V$ in Figure 5b reached ~240 nm which is 40 times larger than ~6 nm at $V_{e}=0\;V$ in Figure 5a. This displacement increment is due to the DC biasing across the electrodes originating from the electret potential. 

**Figure 6:**
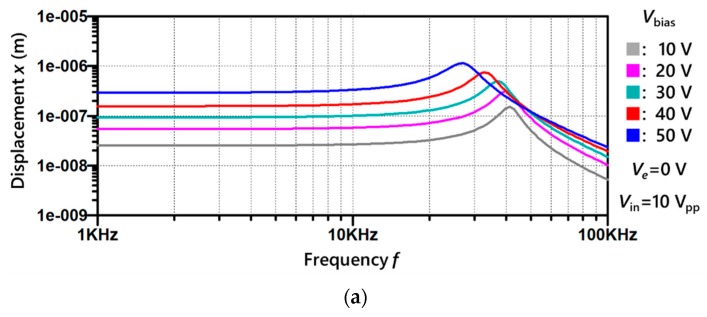

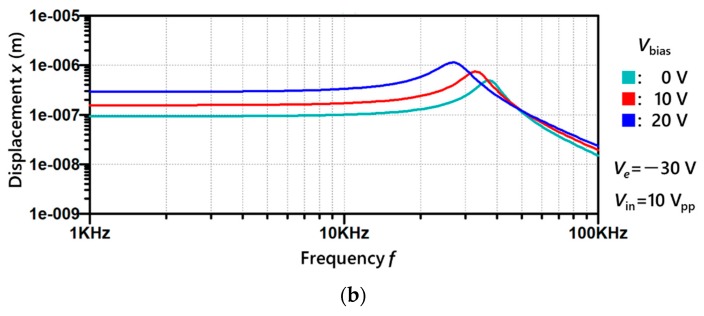
Simulation of dynamic characteristics of gap-closing parallel plate electrode model with electret: (**a**) electret potential $V_{e}=0\;V$; (**b**) electret potential $V_{e}=-30\;V$.

Furthermore, dynamic characteristics of the gap-closing parallel plate electrode model are shown in Figure 6. The electret potentials are $V_{e}=0\;V$ in Figure 6a and $V_{e}=-30\;V$ in Figure 6b, with an equal AC input of $V_{in}=10\;V_{\mathrm{pp}}$. Considering the additional DC biasing, frequency characteristics at $V_{bias}=0\;V,\;10\;V,\;20\;V$ for Figure 6b with an electret potential of $V_{e}=-30\;V$ matches frequency characteristics at $V_{bias}=30\;V,\;40\;V,\;50\;V$ in Figure 6a without any electret biasing. This indicates that the electret potential can be considered as equivalent to external DC biasing, leading to an increment in the displacement.

## 3. Electret-Augmented Out-of-Plane Membrane Actuator

### 3.1. Device Fabrication

**Figure 7:**
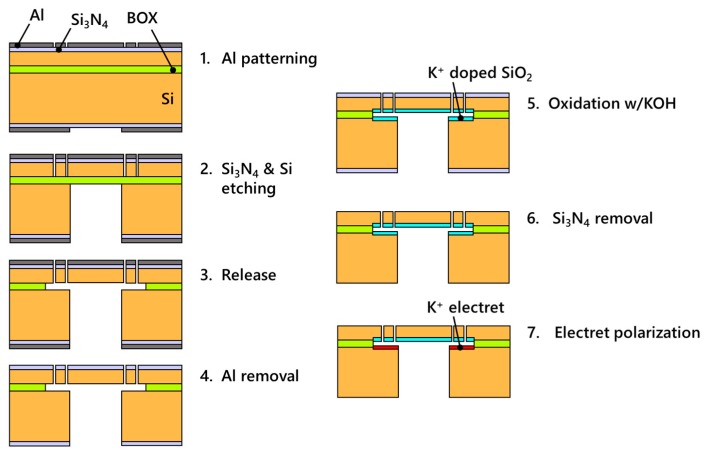
Process flow chart of out-of-plane membrane actuator.

**Figure 8:**
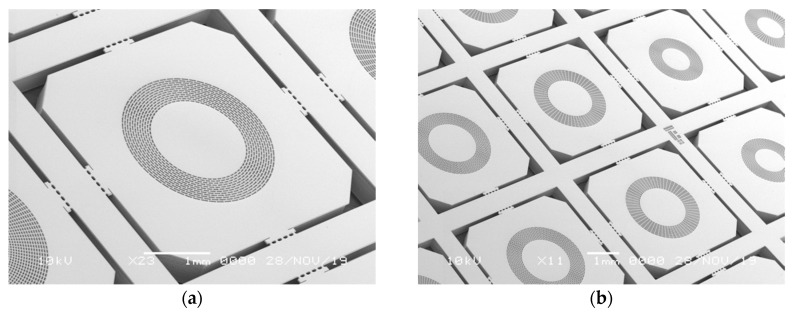
SEM pictures of fabricated out-of-plane membrane actuators: (**a**) individual chip; (**b**) chips on fabricated wafer.

As the parallel plate electrode modeling for first order approximation proved in Section 2.3, electrets augment the outputs of electrostatic actuators by simply incrementing the voltage applied between the electrodes. An electret integrated device with an out-of-plane motion membrane is fabricated to demonstrate this displacement augmentation by electrets. The device fabrication consists of two steps drawn in Figure 7: silicon microfabrication and potassium-ion-electret fabrication. The silicon microfabrication utilized the LOCOS (local oxidation of silicon) method [24] to achieve electrical contact after potassium-ion-electret fabrication. The device was made from an SOI wafer with a 25-μm-thick device layer, a 2-μm-thick BOX layer, and a 500-μm-thick handle. The device layer and the handle were both P-type doped silicon with a 70 nm silicon nitride layer at the surface. First, a circular diaphragm supported by a surrounding mesh structure was etched in the device layer through an aluminum etching mask. The handle silicon beneath this diaphragm was also backside etched. To release the membrane, the underlying BOX layer was etched using an aluminum-ion-saturated silicon dioxide etchant (Silox Vapox III, Transene Company, Inc., Danvers, MA, USA) which does not damage aluminum during silicon dioxide etching. The aluminum mask for silicon etching worked as a protective layer to preserve the silicon nitride at the surface during the long-time BOX etching. Figure 8 are SEM pictures of the fabricated devices.

After finishing the silicon microfabrication, the device was placed in a furnace to thermally grow a layer of potassium ion incorporated silicon dioxide. The device was oxidized for four hours at 1000 °C while exposed to nitrogen bubbled through a 40% potassium hydroxide solution, resulting in a 500-nm-thick oxide layer. The remaining nitride at the surface was removed using carbon tetrafluoride plasma to obtain electrical contact with the silicon layers. Finally, the oxidized device was placed on a silicon heater in a vacuum chamber, and a DC voltage was applied between the device layer and the handle to polarize the oxide facing the inner surface of the SOI handle. As for the potassium-ion-electret method, the electret potential is controlled by the DC voltage applied here and a negatively biased electret will be fabricated. 

### 3.2. Electromechanical Measurements

**Figure 9:**
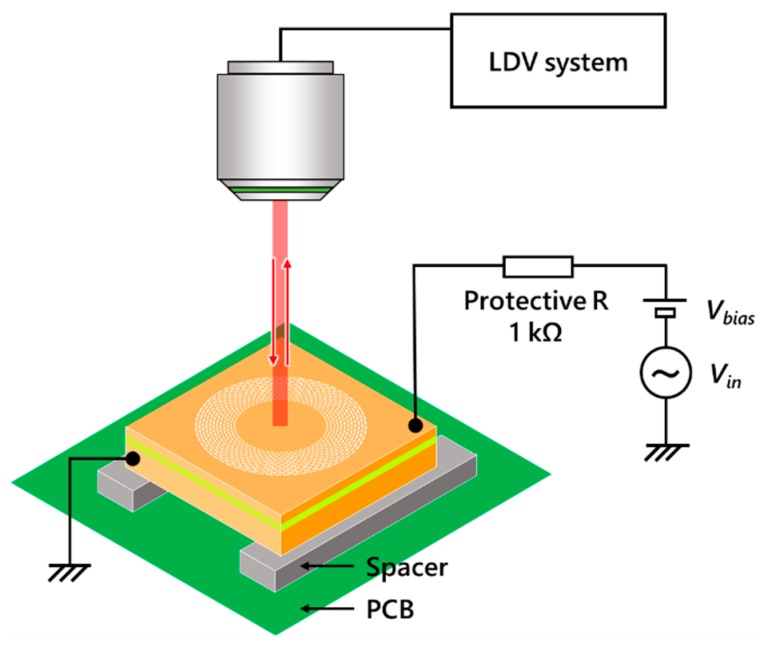
Measurement setup for electromechanical characterization of out-of-plane membrane actuator.

**Figure 10:**
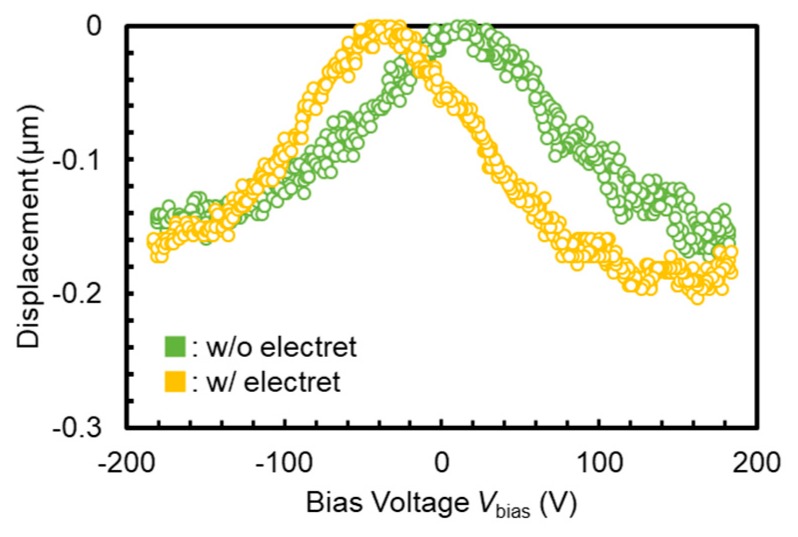
Voltage-displacement characteristics of out-of-plane ϕ=1.5 mm membrane actuator before and after electret fabrication.

An out-of-plane membrane actuator with a membrane diameter of ϕ=1.5 mm was fabricated to verify the displacement augmentation of electret integrated actuators. During the electret fabrication process in Section 3.1, a −50 V bias was applied for the oxide polarization. Electromechanical features of this device were characterized with the measurement setup shown in Figure 9. The displacement of the membrane was measured with a laser doppler vibrometer (MLD-103, NEOARK, Tokyo, Japan) while supplying voltage from a function generator (33500B, Agilent, Santa Clara, CA, USA) for static characteristics, and a servo analyzer (DS-3200, Onosokki, Yokohama, Japan) for dynamic characteristics. Figure 10 shows voltage-displacement characteristics of the device before and after electret fabrication. After electret fabrication, the overall graph shifted by −40 V from the original point. Since voltage was applied to the counterpart electrode of the negatively charged electret, this indicates that the applied voltage cancelled the electret potential at −40 V and consequently the displacement became zero. Compared to the parallel plate electrode model in Section 2.2, a distinct pull-in was not observed since the actuation parts of the actual device are at both ends of a double-clamped beam. 

**Figure 11:**
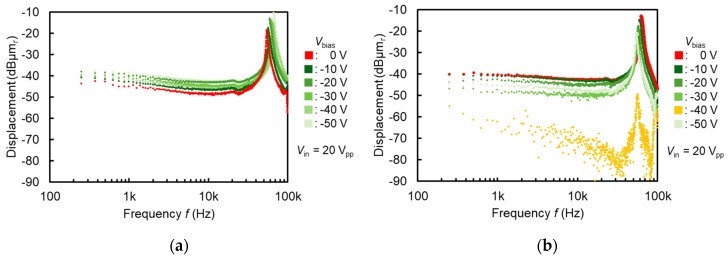
Frequency characteristics of out-of-plane ϕ=1.5 mm membrane actuator: (**a**) before electret fabrication; (**b**) after −40 V electret fabrication. Unit of vertical axis is standardized displacement ${\mathrm{dB}\mu m}_{r}=20\log\frac{X\;\left({\mu m}_{r}\right)}{1\;\left({\mu m}_{r}\right)}$.

Furthermore, frequency characteristics of the device are shown in Figure 11. External DC bias voltages ranging between $V_{bias}=0\sim -50\;V$ were applied to the counterpart electrode of the negatively charged electret with an AC driving voltage of $V_{in}=20\;V_{\mathrm{pp}}$. Figure 11a shows results of the device before the electret fabrication, and Figure 11b shows results of the device after the electret fabrication. In Figure 11b, membrane displacement decreased with larger negative bias voltage, reaching no sufficient output at $V_{bias}=-40\;V$. Along with the voltage-displacement characteristics in Figure 10, a bias voltage equivalent to −40 V was verified to have been supplied from the integrated electret layer. This means that even when the voltage input is limited to the signal $V_{in}=20{\;V}_{\mathrm{pp}}$ without any DC biasing, the displacement reaches $-40\;{\mathrm{dB}\mu m}_{r}$ which originally required a DC biasing of $V_{bias}=-40\;V$ to achieve. 

### 3.3. Acoustic Measurements

**Figure 12:**
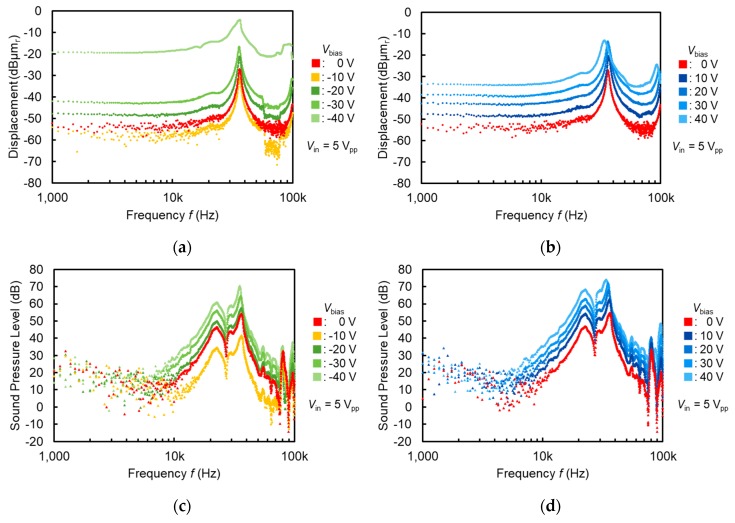
Electromechanical and acoustic characteristics of out-of-plane ϕ=2.0 mm membrane actuator with −10 V biased electret: (**a**) electromechanical characteristics with negative DC biasing; (**b**) electromechanical characteristics with positive DC biasing; (**c**) acoustic characteristics with negative DC biasing; (**d**) acoustic characteristics with positive DC biasing.

For acoustic measurements, a −10 V electret-augmented actuator with an out-of-plane membrane of ϕ=2 mm was fabricated. Electromechanical characteristics of this device are shown in Figure 12a for the negative DC range ($V_{bias}=0\;V\sim -40\;V$) and Figure 12b for the positive DC range ($V_{bias}=0\;V\sim 40\;V$), with an AC driving voltage of $V_{in}=5\;V_{\mathrm{pp}}$. To obtain the acoustic characteristics, a microphone (MI-1271M12, Onosokki, Yokohama, Japan) was placed at a distance of 1.5 cm from the device instead of the laser doppler velocimeter in Figure 9. Figure 12c shows acoustic characteristics for the negative DC range ($V_{bias}=0\;V\sim -40\;V$), and Figure 12d shows characteristics for the positive DC range ($V_{bias}=0\;V\sim 40\;V$). DC biasing of $V_{bias}=-10\;V$ cancelled out the electret potential, resulting in the minimum displacement and sound pressure level in Figure 12a,c. For positive biasing in Figure 12b,d, the potential enhancement led to the increase in both the membrane displacement and the sound pressure level. Without any DC biasing but only with the signal input of $V_{in}=5{\;V}_{\mathrm{pp}}$, the sound pressure level reached 50 dB at maximum. However, a number of peaks were observed during the acoustic measurements in Figure 12c,d, which did not appear at the electromechanical measurements in Figure 12a,b. Seemingly, this occurred due to the divided vibration of the membrane by the interference or the phase shift between the circular diaphragm and the surrounding meshed actuating structure. Also, since silicon dioxide remained only on one side of the diaphragm, residual stress applied strain on the membrane which also leads to a divided vibration. 

## 4. Discussion

As mentioned in the introduction, compared to other types of actuators of different driving principles, electrostatically driven MEMS actuators are compatible with wireless electronics in the sense that capacitors do not consume large current which shortens battery lives. Thus, the remaining high voltage was the issue in consuming limited power sources in wireless electronics. Although the current consumption of the actuator itself does not affect battery lives, the current and power consumed in the driving circuit for the actuator does. Given the AC signal from the module where the actuator is used in, a DC-DC converter is otherwise necessary to provide high DC voltage. Generally, DC-DC converters consume large power of hundreds of milliwatts, which is a non-negligible amount for portable batteries or energy harvesters that supply power to wireless electronics. By supplying the DC bias voltage from electrets, an amount of −40 V demonstrated in this paper, the DC-DC converter will no longer be necessary. Hence, substituting the external DC bias to an electret potential contributes to reducing the overall power consumption of the device system.

## 5. Conclusions

Device integrated electrets were suggested to replace external DC power supplies which MEMS electrostatic actuators conventionally required. Focusing on the electret property, a −40 V biased electret layer was fabricated at the inner surface of a SOI where electrodes faced a 2 μm gap, owing to the high compatibility of the potassium-ion-electret method and silicon micromachining. For application, a −10 V electret-augmented actuator with an out-of-plane motion membrane reached an acoustic output of 50 dB maximum. Although these devices were designed to exhibit a flat frequency characteristic within the audible range for possible usage in ear canals, the resonance frequency can be shifted for different usages by controlling device parameters such as the diameter of the membrane, width, and filling ratio of the meshed part, or the thickness of the SOI. In addition to usages as acoustic transducers, electret biased devices will further contribute to the enhancement of low-voltage, low-power driven MEMS electrostatic actuators.

## Figures and Tables

**Table 1:** Parameters used for gap-closing parallel plate electrode model simulation

Parameter	Symbol	Value
Spring constant	*k*	8000 N/m
Viscous damping coefficient	*c*	5 × 10^−3^
Mass	*m*	1.15 × 10^−7^ kg
Electrode gap	*g*	2 μm
Electrode area	*S*	2 mm^2^
Electret thickness	*t*	500 nm
Location of electret charge	*r*	250 nm
